# Circular Dichroism without Absorption in Isolated Chiral Dielectric Mie Particles

**DOI:** 10.1021/acsphotonics.5c02076

**Published:** 2026-02-09

**Authors:** Rafael S. Dutra, Felipe A. Pinheiro, Diney S. Ether, Cyriaque Genet, Nathan B. Viana, Paulo A. Maia Neto

**Affiliations:** † 133629 LISComp-IFRJ, Instituto Federal de Educação, Ciência e Tecnologia, Rua Sebastião de Lacerda, Paracambi 26600-000, Brasil; ‡ Instituto de Física, 28125 Universidade Federal do Rio de Janeiro, Caixa Postal 68528, Rio de Janeiro, Rio de Janeiro 21941-972, Brazil; § Institut de Science et d’Ingénierie Supramoléculaires, Université de Strasbourg, CNRS, UMR, F-67000, Strasbourg 7006, France

**Keywords:** circular dichroism, chirality, mie scattering, nonparaxial optics, polarimetry, dielectric nanoparticles

## Abstract

We demonstrate that an effect phenomenologically analogous to circular dichroism can arise even for dielectric and isotropic chiral spherical particles. By analyzing the polarimetry of light scattered from a chiral, lossless microsphere illuminated with linearly polarized light, we show that the scattered light becomes nearly circularly polarized, exhibiting large nonresonant values of the Stokes parameter *S*_3_ for a broad range of visible frequencies. This phenomenon occurs only in the Mie regime, with the microsphere radius comparable to the wavelength, and provided that the scattered light is collected by a high-NA objective lens, including nonparaxial Fourier components. Altogether, our findings offer a theoretical framework and motivation for an experimental demonstration of a novel chiroptical effect with isolated dielectric particles, with potential applications in enantioselection and characterization of single microparticles, each and every one with its own chiral response.

## Graphical abstract

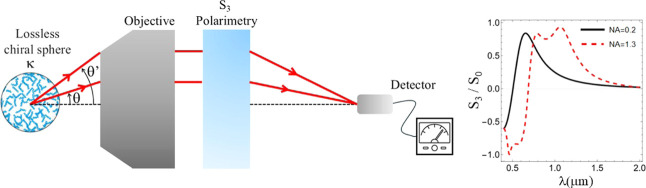

## Introduction

An object is considered chiral if it has nonsuperposable mirror images, i.e., two enantiometers. The separation of chiral enantiomers is a significant scientific and technological challenge with broad, multidisciplinary applications.^1–3^ Chirality also shows up in the optical properties of materials in a very characteristic way so that the chiroptical response provides one of the most direct and effective means for analyzing chiral systems. Indeed, chiral objects exhibit differential absorption of left- and right-handed circularly polarized light, known as circular dichroism (CD).^1–3^ Additionally, chiral systems can rotate the plane of incident linearly polarized light in a direction determined by their handedness, a phenomenon known as optical rotatory power.^1–3^

CD spectroscopy is one of the most traditional methods employed for the enantioselective detection of chiral molecules.^4^ The resulting CD spectra are unique to a molecule’s specific conformation, with the sign of the signal indicating the enantiomer’s handedness. However, the intrinsically weak chiroptical signals fundamentally limit the sensitivity of CD spectroscopy, so that it typically probes bulk samples. An enhanced sensitivity was achieved with nonlinear resonant phase-sensitive microwave spectroscopy.^5^ The advent of nanophotonics and plasmonics has led to the development of various strategies to enhance CD, thereby enabling more efficient chiral sensing.^6,7^ The limitations imposed by intrinsically weak CD signals become particularly pronounced in single-molecule sensing, requiring special techniques in the case of individual molecules, for instance, fluorescence-detected CD,^8^ as well as in the characterization of larger, isolated chiral microparticles, which are promising platforms for applications in nanophotonics,^9–14^ such as enantioselection via optical forces.^15–22^ To circumvent these limitations, different strategies that include substrate-assisted CD,^23^ extrinsic chirality,^24^ imaging techniques,^25,26^ and plasmonic materials have been employed to enhance the weak chiroptical response of single chiral nanoparticles.^24,27–29^ Indeed, thanks to the strong interaction between light and free electrons, chiral plasmonic nanoparticles exhibit distinctive resonances that enable the experimental observation of single-particle CD spectroscopy.^28,30,31^ This technique allows for the detection of CD in individual chiral nanoparticles by measuring differences in extinction, scattering, or absorption between left- and right-circularly polarized light enabling enantiomeric recognition.^28,30^ In addition to designing plasmonic particles with chiral geometries,^32,33^ other strategies to enhance chiroptical effects include synthesizing plasmonic systems in the presence of chiral molecules or under conditions breaking mirror symmetry,^34^ chiral optical cavities,^35–37^ and photothermal approaches.^38^ By enhancing the chiral optical response, these strategies allow for enantioselection and chiral characterization on the scale of single nanoparticles. However, since these approaches typically rely on the excitation of plasmonic resonances, the enhancement of chiroptical properties in single nanoparticles often comes at the expense of high losses and limited frequency bandwidths.^39–41^

In this context, the development of alternative mechanisms for probing the chiroptical response of individual chiral nanoparticles without relying on plasmonic effects has become increasingly important. This need is further underscored by the recent developments and applications of all-dielectric chiral nanosystems, such as optical cavities for enhanced chiral sensing.^42,43^ These cavities can be Mie particles that support high quality factor resonances, tailored to assist and facilitate chiral sensing, chiral transfer, and enantioselection.^44–46^ Chiral sensing of spherical analytes can be either relevant for naturally occurring chiral materials, such as, for instance, limonene emulsions in water, in which spherical droplets form due to interfacial surface tension,^47^ or nanostructured chiral Mie particles,^33^ with potential applications in the emerging field of Mie-tronics.^48^ Very recently, growing interest in the chiral optical response of single chiral Mie spheres has motivated the development of novel chiroptical and enantioselective techniques for these particles.^49^

Building on these motivations, the present study reveals a novel chiroptical response in single, lossless, and isotropic chiral Mie microspheres, which is phenomenologically manifested as the well-known CD observed in absorbing media. Specifically, we demonstrate that linearly polarized light scattered by such particles—and collected using a high-numerical-aperture (numerical aperture (NA)) objective lens—becomes nearly circularly polarized, leading to enhanced, nonresonant values of the Stokes parameter *S*_3_, which quantifies the degree of circular polarization of electromagnetic radiation,^50–52^ across a broad range of visible frequencies, in contrast to cases assisted by plasmonic resonances. We show that this effect arises intrinsically from the nonparaxial Fourier components of the scattered light and the underlying Mie scattering regime. These findings not only reveal a CD-like response in lossless particles but also open new avenues for applying Mie-tronics to chiral sensing, chiral characterization, and enantioselective technologies.^48^

## Theoretical Model

In this section, we describe the model to achieve imaging of chiral, homogeneous Mie microspheres after propagation of the field scattered into the forward hemisphere through the microscope objective. The incident illumination on the microsphere is described as a plane wave of wavelength λ propagating in water with a wave vector magnitude *k*_w_ = 2π*n*_w_/λ and linearly polarized along the x̂ direction, represented by the electric field

1 $$E=E_{0}e^{i\left(kwz-\omega t\right)}\mathrm{x̂}$$

where *n*_w_ is the refractive index of water. The setup is schematically depicted in Figure 1a.

**Figure 1:**
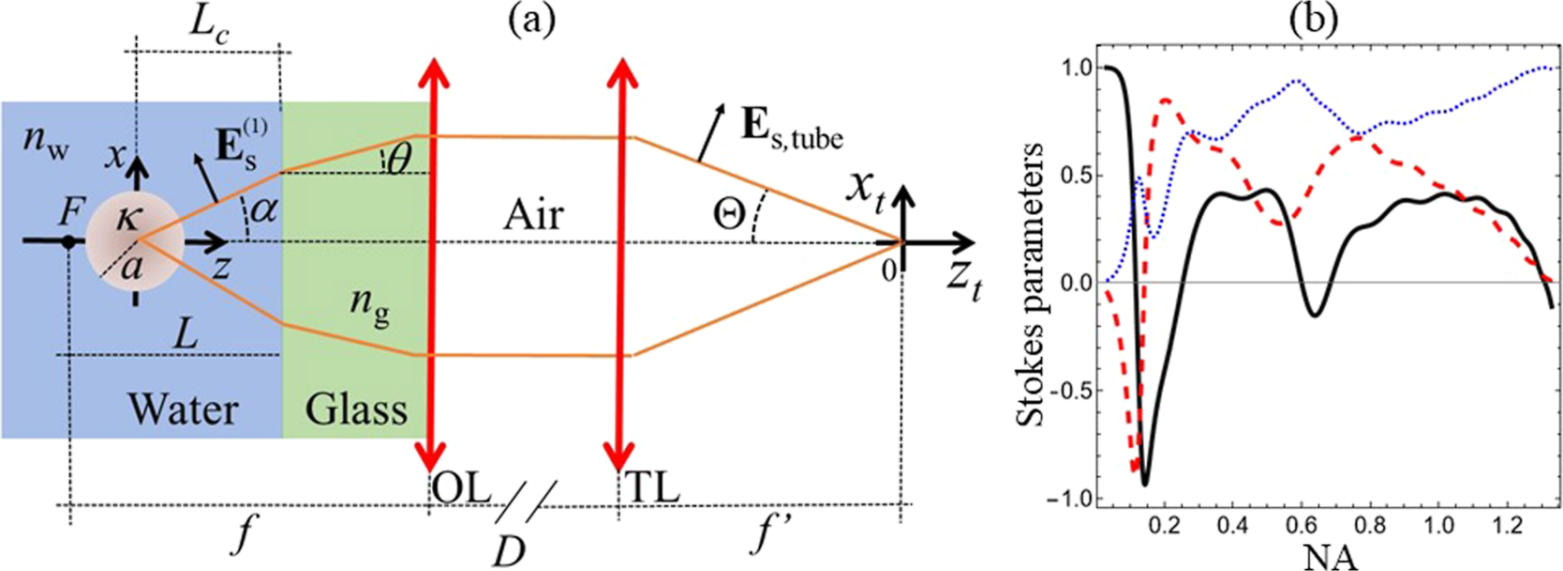
(a) Imaging configuration in an optical microscope with the collection of light scattered in the forward hemisphere. The incident illumination, described by a plane wave, is scattered by the microsphere, with radius *a* and chirality parameter κ. Then, the illumination is collected by the objective lens OL and finally focused by the tube lens TL with respective focal lengths *f* and *f*′. (b) Stokes parameters *S*_1_ (black solid line), *S*_2_ (red dashed line), and *S*_3_ (blue dotted line), normalized by the Stokes parameter *S*_0_, as functions of the NA of the objective lens, for a chirality parameter set to κ = – 0.02 and a wavelength λ = 0.464 μm.

We assume that the microsphere is composed of a homogeneous and isotropic chiral material in which the electric field and the magnetic field strength **H** are coupled to the displacement field **D** and the magnetic flux density **B** according to the following constitutive relations:^53^

$$\left(\begin{array}{l} D\\ B \end{array}\right)=\left(\begin{array}{ll} \epsilon_{0}\epsilon & i\kappa /c\\ i\kappa /c & \mu_{0}\mu \end{array}\right)\left(\begin{array}{l} E\\ H \end{array}\right)$$ 2

where ϵ and μ are the relative permittivity and permeability, $c=1/\sqrt{\epsilon_{0}\mu_{0}}$ is the speed of light in vacuum, and κ is the pseudoscalar defined as the chirality parameter or chiral index of the medium (Pasteur parameter), which couples the electric and magnetic fields. From a practical point of view, κ is proportional to the specific rotation ${\left[\alpha \right]}_{\lambda }^{T}$ with $\kappa =\lambda \left[dm\right]\rho \left[g/mL\right]{\left[\alpha \right]}_{\lambda }^{T}/360{}^{\circ}$ for a lossless material of density ρ.^54^

We expand the incident electric field (1) as well as the corresponding magnetic field as superpositions of spherical multipole waves written in terms of Debye potentials.^55,56^ In the circular polarization basis, the scattering matrix of the chiral microsphere is diagonal in the representation defined by the electric (*E*) and magnetic (*M*) multipoles. Hence, we expand the incident polarization vector $\hat{x\;}=\left({\mathrm{\varepsilon ̂}}^{+}+{\mathrm{\varepsilon ̂}}^{-}\right)/\sqrt{2}$ in the circular polarization basis ε̂ ^σ^ = ( x̂ + iσ ŷ )/ $\sqrt{2}$ , with σ = ± 1 denoting the helicity,^57^ and then solve for the scattered field at position **r**(*r*, Θ, Φ) written in terms of spherical coordinates for each helicity component. The scattered Debye potentials for helicity σ associated with electric and magnetic multipoles are given by^19,58,59^

3 $$\Pi_{s,\sigma }^{E}\left(r,\Theta ,\Phi \right)=-\frac{\sigma E_{0}}{k_{W}}\sum_{j=1}^{\infty }i^{j+1}A_{j}^{\sigma }\sqrt{\frac{4\pi \left(2j+1\right)}{j\left(j+1\right)}}Y_{j}^{\sigma }\left(\Theta ,\Phi \right)h_{j}^{\left(1\right)}\left(k_{w}w\right)$$

and

4 $$\Pi_{s,\sigma }^{M}\left(r,\Theta ,\Phi \right)=-\frac{H_{0}}{k_{W}}\sum_{j=1}^{\infty }i^{j}B_{j}^{\sigma }\sqrt{\frac{4\pi \left(2j+1\right)}{j\left(j+1\right)}}Y_{j}^{\sigma }\left(\Theta ,\Phi \right)h_{j}^{\left(1\right)}\left(k_{w}r\right)$$

where $Y_{j}^{\sigma }$ are the spherical harmonics. The electric *E*_0_ and magnetic *H*_0_ amplitudes are related by *H*_0_ *=* $\sqrt{\varepsilon_{w}{/\mu }_{0}}$ *E*_0_, where ε_*w*_ and μ_0_ are the electric permittivity of water and the magnetic permeability of vacuum, respectively. Physically, the Hankel functions $h_{j}^{\left(1\right)}\left(k_{w}r\right)$ describe the outgoing behavior of the scattered spherical waves. The expressions for the effective Mie scattering coefficients $A_{j}^{\sigma }$ and $B_{j}^{\sigma }$ for a chiral sphere of radius *a* are presented in Section 1 of the Supporting Information. The scattered electric field for a given helicity σ, $E_{s}^{\sigma }=E_{s}^{\sigma ,E}+E_{s}^{\sigma ,M}$ , can be decomposed into electric $E_{s}^{\sigma ,E}=\left(-i\right)\nabla \times L\left(\Pi_{s,\sigma }^{E}\right)$ and magnetic $E_{s}^{\sigma ,M}=\mu_{0}/\varepsilon_{w}k_{w}L\left(\Pi_{s,\sigma }^{M}\right)$ multipoles by applying vector operators on the Debye potentials. The vector operators are expressed in terms of the orbital angular momentum operator **L** = – *i* **r** × ∇. To describe light propagation of the scattered field through the optical system, we extend the approach of ref 60 and expand the scattered spherical waves into plane waves employing the Weyl integral representation^61–64^

5 $$Y_{j}^{\sigma }\left(\Theta ,\Phi \right)h_{j}^{\left(1\right)}\left(k_{w}r\right)=\frac{i^{-j}}{2\pi }\int_{0}^{2\pi }d\beta \int_{C}d\alpha \sin\alpha {\;Y}_{j}^{\sigma }\left(\alpha ,\beta \right)e^{ik_{w}\left(\alpha ,\beta \right)\cdot r}.$$

The direction of the wavevector **k**_*w*_(α, β) is determined by the spherical angles (α, β). The integration contour *C* is selected^63^ to account for both evanescent waves (imaginary values of α) and homogeneous waves that propagate in the forward hemisphere *z* > 0 (*k*_*wz*_ > 0). As a result, the scattered field expanded into plane waves in the aqueous solution reads

$$E_{s}^{\left(w\right)}\left(r\right)=-\frac{E_{0}}{8\pi }\underset{\sigma =-1,+1}{\sum }\overset{\infty }{\underset{j=1}{\sum }}\left(2j+1\right)\overset{2\pi }{\underset{0}{\int }}d\beta \int_{C}d\alpha \sin\alpha +\left[\left(A_{j}^{\sigma }+B_{j}^{\sigma }\right)d_{\sigma ,\sigma }^{j}\left(\alpha \right)e^{i\sigma \beta }-\left(A_{j}^{\sigma }+B_{j}^{\sigma }\right)d_{-\sigma ,\sigma }^{j}\left(\alpha \right)e^{-i\sigma \beta }\right]\left(\mathrm{\vartheta ̂}+i\sigma \mathrm{\varphi ̂}\right)e^{ik_{w}\left(\alpha ,\beta \right)\cdot r}.$$ 6

It is defined in terms of the matrix elements of finite rotations d_m′_, _m_^j^(α)^65^ with *m* = *m*′ = σ for terms that conserve helicity, and *m* = – *m*′ = σ for the contributions that flip helicity upon scattering by nondual Mie spheres.^66–69^

The imaging setup consists of an optical microscope where the scattered light is initially collected by an oil immersion objective with NA > 1, focal length *f*, and aperture angle θ_0_ = arcsin­(NA/*n*_*g*_), where *n*_*g*_ is the refractive index of the glass, as depicted in Figure 1a. Each Fourier component of the scattered field $E_{s}^{\left(w\right)}\left(r\right)$ given by ([Disp-formula eq6]) is characterized by its wave vector **k**_w_(α, β). As the Fourier components propagate away from the microsphere, they first refract at the interface between the sample chamber and the glass slide, as shown in Figure 1a. In addition to a reduction of amplitude, the spherical aberration phase^70–73^

7 $$\Phi_{g-w\left(\theta \right)}=k_{g}\left(-L\cos\theta +{NL}_{c}\cos\alpha \right)$$

arises from refraction at this planar water–glass interface. Here, θ = arcsin­(*n*_w_ sin α/*n*_*g*_) is the refraction angle in the glass medium, *N* = *n*_w_/*n*_*g*_ is the relative refractive index for the interface, and *k*_*g*_ = 2π*n*_*g*_/λ is the wavenumber for propagation in glass. The aberration function Φ_g‑w_(θ) scales with the lengths *L* and *L*_*c*_ representing the positions of the focal plane and of the microsphere center of mass, respectively, both relative to the water–glass interface.

After refraction, the scattered light is collected by the microscope objective and then propagates through the tube lens (focal length *f*′) of a low NA, where it is eventually focused on the camera. The same process occurs for the field associated with the illumination that propagates toward the tube lens to ultimately interfere with the field scattered by the microsphere at the camera position.

The scattered field at the focus of the tube lens is derived after considering the propagation through the imaging system (see Supporting Information) and reads

8 $$E_{s,tube}=\frac{E_{0}}{4}\frac{f}{n_{g}f^{\prime }}e^{{ik}_{g}fe^{{ik}_{0}\left(D+f^{\prime }\right)}}\sum_{j=1}^{\infty }\sum_{\sigma =-1}^{+1}\left(2j+1\right)\left(A_{j}^{\sigma }+B_{j}^{\sigma }\right)\int_{0}^{\theta_{0}}d_{1,1}^{j}\left(\alpha \right)f\left(\theta \right)d\theta \left(\mathrm{x̂}+i\sigma ŷ\right),$$

with $f\left(\theta \right)=\left(\sin\theta /\cos\alpha \right){\left(\cos\theta \right)}^{3/2}T⊥\left(\theta \right)e_{g-w}^{i\Phi }\left(\theta \right)$ . In addition, *D* is the distance between the objective and the tube lens, *k*_0_ = 2π/λ is the wavenumber in air, and *T*_⊥_(θ) is the Fresnel refraction amplitude for the water–glass interface. The total electric field is given by the coherent superposition **E**_tot,tube_ = **E**_in,tube_ + **E**_s,tube_, where the expression of the incident electric field **E**_in,tube_ can be found in the Supporting Information.

We perform polarimetry of the detected total field using the Stokes parameters *S*_0_, *S*_1_, *S*_2_, and *S*_3_ ^74–78^ written as functions of the total electric field components, namely: $S_{0}=E_{x}E_{x}*+E_{y}E_{y}*$ that represents the intensity of the detected total field; $S_{1}=E_{x}E_{x}*-E_{y}E_{y}*$ that gives the amount of horizontal and vertical linear polarizations; $S_{2}=E_{x}E_{y}*+E_{y}E_{x}*$ that describes the amount of diagonal polarizations along the 45° and 135° directions; and $S_{3}=i\left(E_{x}\;E_{y}\;*-E_{y}\;E_{x}\;*\right)$ that accounts for the amount of circular polarization in the left and right directions. In the next section, we calculate *S*_1_, *S*_2_, and *S*_3_ normalized by parameter *S*_0_.

## Results and Discussion

In the following, we consider realistic values for the optical system parameters: tube lens focal length *f*′ = 20 cm, and glass refractive index *n*_*g*_ = 1.51. In most examples, we take NA = 1.3 for the objective lens and obtain its focal length *f* from the typical magnification M = 100× of the objective as *f* = *n*_*g*_ *f*′/M = 0.5 cm.^60^ We account for the dispersion of water encoded in the refractive index formula *n*_w_ = 1.3219 + 3.631 × 10^–3^/λ­[μm] and set ϵ = 2.1 for the relative electric permittivity of the microsphere.^18^ We also consider the microsphere centered on the optical axis and touching the water–glass interface (*L*_*c*_ = *a*) and take the focal plane at the water–glass interface (*L* = 0), to reduce the spherical aberration arising from refraction at this interface.

Figure 1b highlights one of the key findings of this work, namely, the fact that the Stokes parameter *S*_3_ not only can be nonvanishing but also may reach large values for lossless spherical particles, particularly for large (nonparaxial) values of the NA of the objective employed in the proposed imaging setup depicted in Figure 1a. *S*_3_ usually describes the well-known effect of CD, which is the differential absorption of left- and right-handed circularly polarized light.^30^ To investigate how the nonparaxial nature of the optical system influences the detected polarization, we analyze the variation of the Stokes parameters with the objective NA in Figure 1b. We choose the wavelength λ = 0.464 μm and consider a microsphere of radius *a* = 1.5 μm and chirality parameter κ = – 0.02. The detected polarization is approximately left-handed circular (σ = +1) with *S*_3_ ≈ + 1 when NA = NA_max_ = 1.3. As one changes the NA, we consider a fixed value for the radius of the objective back aperture in order to collect the same power in all cases. As a consequence, the focal length changes according to *f* = *f*_max_ NA_max_/NA, where *f*_max_ = 0.5 cm is the focal length for NA_max_ = 1.3 as mentioned earlier.

In the paraxial limit, which corresponds to detecting a single forward plane wave (NA → 0), the horizontal linear polarization of the incident field is approximately conserved during scattering, and therefore, *S*_1_ → 1, as shown in Figure 1b. As the NA increases, the horizontal polarization rotates counterclockwise, passing through states close to maximum diagonal and vertical polarizations around NA ∼ 0.1 and NA ∼ 0.15, respectively. For higher values of NA, while the degree of circular polarization, represented by the Stokes parameter *S*_3_, increases nonmonotonically, the parameters *S*_1_ and *S*_2_ reduce in a nontrivial manner until they reach zero for NA_max_ = 1.3, while the detected beam reaches an approximately pure state of circular polarization.

Overall, Figure 1b makes evident the importance of considering high NA and the corresponding large off-axis scattering angles in order to observe a nonvanishing *S*_3_ for dielectric chiral particles. Indeed, for small NA, which is typically the case of standard CD spectrometers,^28^ not only is *S*_3_ small, but also it is smaller than *S*_2_. Indeed, rotatory power is the most appropriate way to probe the chirality of dielectric chiral particles in the paraxial regime. In contrast, as one increases NA, *S*_3_ significantly overcomes *S*_2_, suggesting that in this case, addressing *S*_3_ should facilitate the characterization of the optical response of lossless chiral particles. We verified that this conclusion holds even in the presence of typical values of absorption for dielectric materials.

We note that we find a nonvanishing optical rotatory power, quantified by the Stokes parameter *S*_2_ shown in Figure 1b, despite the fact that the scatterer is not dual. This result does not contradict the general conditions for optical rotation in chiral media, which state that, in addition to the breaking of spatial inversion symmetry, dual symmetry is required when considering a single, nonforward scattering direction.^79–81^ Indeed, the detected signal in our setup, depicted in Figure 1a, results instead from the coherent superposition of all scattering directions collected by the objective lens.

It is also important to emphasize that our results are consistent with the conservation law for optical chirality.^82^ Indeed, we find that the total field (incident + scattered) carries a net-zero chirality flux through a Gaussian spherical surface enclosing the Mie scatterer, provided that the host medium is nonabsorbing (see Section 3 of the Supporting Information). This result is consistent with nonzero values of *S*_3_ obtained in the considered detection geometry, in which scattered Fourier components are collected only within the forward hemisphere region delimited by the objective NA.

While the results shown in Figure 1b are valid for particular values of the chirality parameter κ and of the incident wavelength λ, we explore in the color maps of *S*_3_ shown in Figure 2, the full parameter space defined by κ and λ. The analysis of Figure 2 confirms that Mie scattering by a lossless chiral particle can exhibit large values of *S*_3_ over a broad range of values of κ and for a wide range of visible frequencies, which mimics the CD effect. Our findings challenge this traditional scenario, showing that an analogue to CD, associated with a nonvanishing value of *S*_3_, may also exist for lossless chiral particles provided the Mie regime is met. Indeed, Figure 2a, in which *S*_3_ is calculated for the smallest sphere’s radii *a*, show that *S*_3_ is negligible in the dipolar regime (λ ≫ *a*). The right-hand side of Figure 2b also indicates that *S*_3_ goes to zero for large wavelengths. In the opposite limit of geometrical optics (λ ≪ *a*), *S*_3_ is also very small, as shown in a more evident way in the left-hand-side (smallest values of λ) of Figure 2d, which corresponds to the largest value of *a* shown in the figure. Altogether these results highlight the importance of addressing the Mie regime (λ ∼*a*) in order to achieve large values of *S*_3_ even for single, lossless chiral spherical particles, unveiling an effect that is the analogue of CD for all-dielectric chiral particles. Remarkably, Figure 2 also demonstrates that *S*_3_ may change the sign by varying the incident wavelength without changing the sign of κ. This effect does not occur in the dipolar regime (see Figure 2a) and emerges only in the Mie regime, as the majority of important results in this work.

**Figure 2:**
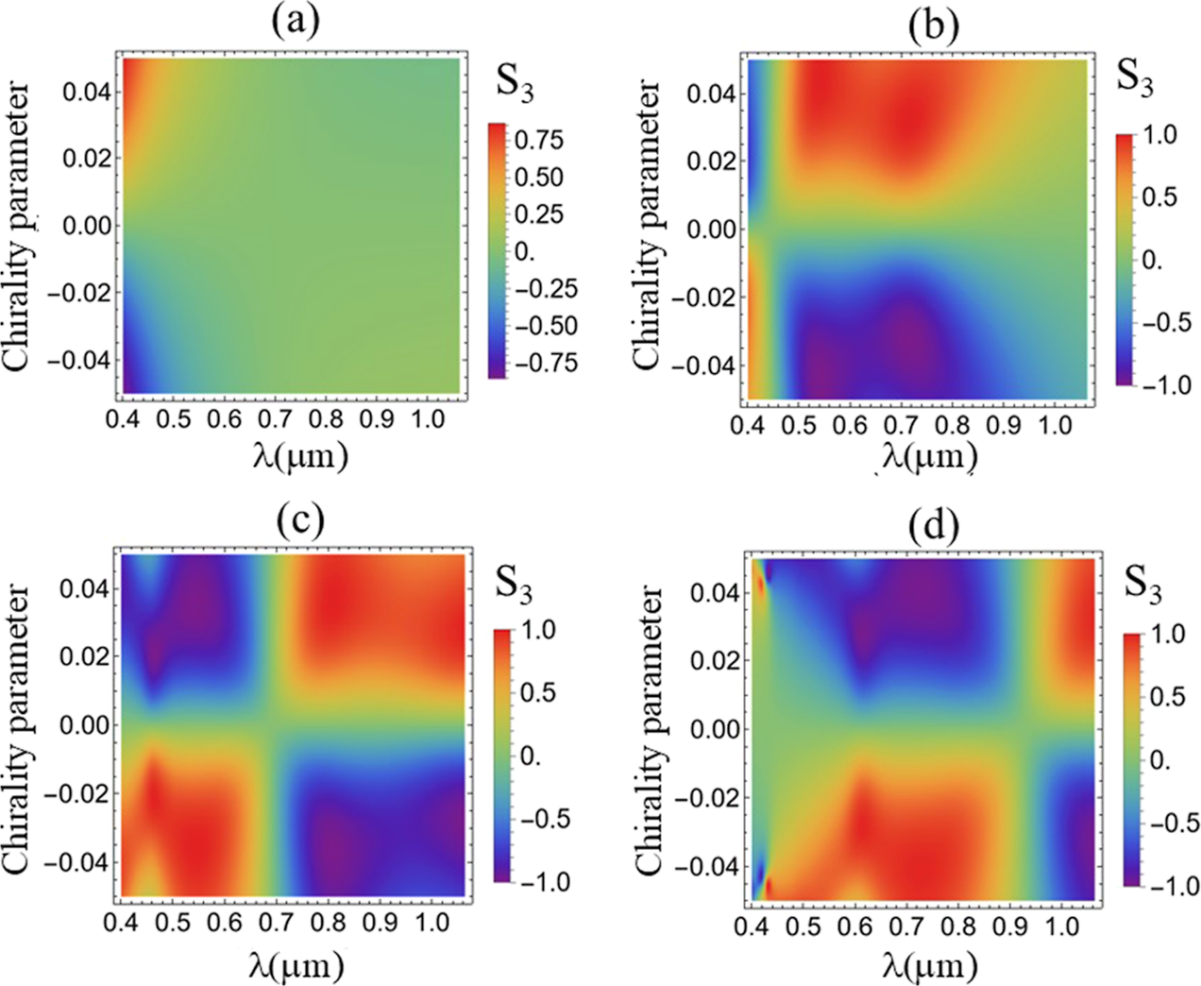
Color maps of the Stokes parameter *S*_3_, normalized by the parameter *S*_0_, as a function of the illumination wavelength λ and the chirality parameter κ, for different values of microsphere radius (a) *a* = 0.5 μm, (b) *a* = 1.0 μm, (c) *a* = 1.5 μm, and (d) *a* = 2.0 μm. The value of the NA is NA = 1.3.

As a matter of consistency, in Figure 2, the polarimetry of the total detected field in the focal plane of the tube lens shows that in the limit of an achiral microsphere (κ → 0), *S*_3_ → 0 and *S*_1_ → 1. In this case, the helicities σ = ± 1 are not unbalanced during detection, and the total detected polarization is similar to the initial linear polarization x̂ . For achiral microspheres, both helicities of the Fourier components, collected along the optical axis in the focal plane of the tube lens, are scattered with the same amplitude (*a*_*j*_ + *b*_*j*_) (see Section 2 of the Supporting Information), thus reflecting the conservation of polarization state in this type of detection. In contrast, the chirality of the microsphere induces an unbalance of helicities during detection. Indeed, according to Figure 2, the degree of circular polarization of the total detected field increases, and becomes fully circularly polarized when *S*_3_ = ± 1, as the absolute value of the chirality parameter increases in certain wavelength ranges.

It is important to emphasize that the calculation of the Stokes parameters in our detection geometry involves the coherent superposition of the Fourier components that are scattered in different directions and that interfere coherently not only with the incident field but also between themselves. In Figure 3, we show the differential Stokes parameter $dS_{3}/d\Omega \;\left(\theta \right)$ (*d*Ω being the solid angle of a thin conical angular shell) associated with the field of a single, individual scattered conical shell of plane waves superimposed with the incident field as a function of the scattering angle θ. For example, note that in Figure 3c, $dS_{3}/d\Omega \;\left(\theta \right)$ is positive for the vast majority of values of θ so that its integral over θ must be clearly positive as well. In contrast, the actual value of *S*_3_ is negative, as shown in Figure 2c, at λ = 0.9 μm and for the parameters corresponding to Figure 3c. Indeed, the integral of $dS_{3}/d\Omega$ (θ) represents an incoherent sum of intensities emerging from the polarimeter whereas our calculations are based on a coherent superposition of all scattered single wave components. As a result, altogether these findings show the crucial role of the coherent superposition and interference of different scattered plane wave components in the sign change of Stokes parameter *S*_3_ shown in Figure 2c.

**Figure 3:**
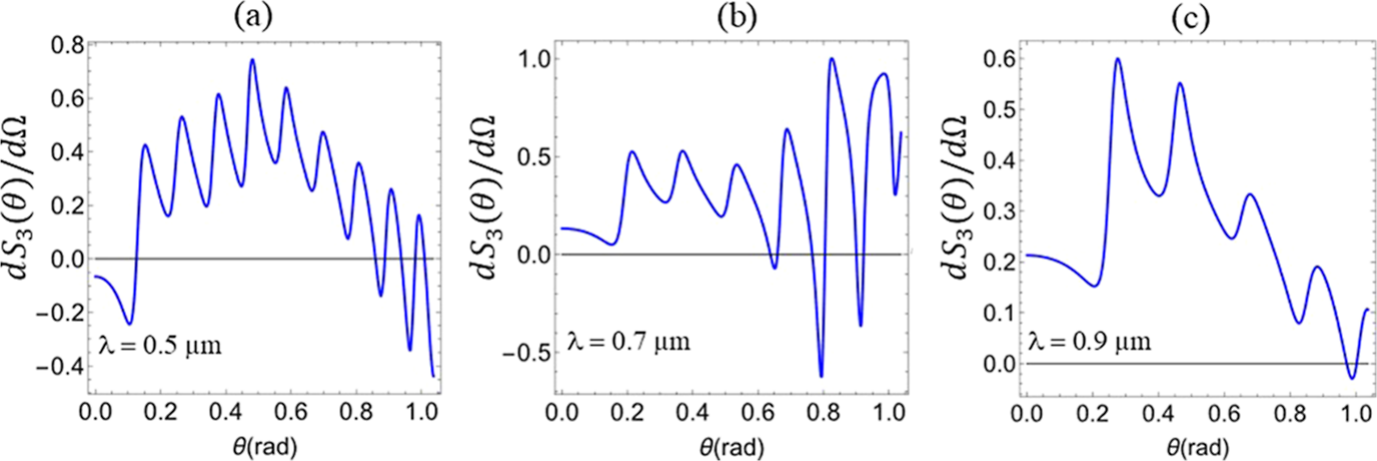
Differential Stokes parameter $dS_{3}/d\Omega \;\left(\theta \right)$ (normalized by *S*_0_), associated with the field of an individual conical shell of scattered plane waves superimposed to the illumination field, as a function of the scattering angle θ for different wavelengths: (a) λ = 0.5 μm, (b) λ = 0.7 μm, and (c) λ = 0.9 μm. We consider a microsphere of radius *a* = 1.5 μm and chirality parameter κ = – 0.02.

For the value of the microsphere radius *a* = 1.5 μm and wavelength λ = 0.464 μm, we study in Figure 4a the dependence of the Stokes parameters on the chirality parameter κ. Interestingly, in this case, |*S*_3_| is larger than |*S*_2_|, even for very small values of κ. This result shows that for a dielectric particle with chirality parameters of the order of naturally occurring materials, the CD-like effect encoded in *S*_3_ may overcome the optical rotatory power, related to *S*_2_. Figure 4a also shows that the detected polarization evolves from a state of horizontal linear polarization (*S*_1_ = 1), for an achiral microsphere (κ = 0), until it reaches maximum circular polarization states with *S*_3_ = ± 1, for chirality parameters close to κ ≈ ∓ 0.02, respectively. Figure 4a reveals that in the vicinities of κ = 0, *S*_3_ exhibits a linear dependence on κ, which allows one to estimate the sensitivity of Stokes parameter *S*_3_ required to determine small chirality parameters. Indeed, Figure 4b shows that |δ*S*_3_/δκ| ≈ 10^2^ so that, considering that the typical sensitivity of state-of-the-art CD spectrometers is of the order *S*_3_/*S*_0_ ≈ 10^–3^,^83^ one could detect chirality parameters as small as |δκ| ≈ 10^–5^. This result opens up the possibility of characterizing the chirality parameter of isolated microdroplets of naturally occurring chiral oils dispersed in an aqueous phase (microemulsions).^47,84^ For instance, limonene oil (density ρ = 0.86 g/mL) has a specific rotation [α]_λ_^T^ = 159.4° ^85,86^ at a temperature of 20 °C when illuminated with the wavelength λ = 464 nm. The corresponding chirality parameter is κ = 2 × 10^–6^, which is close to the sensitivity limit derived from Figure 4b.

**Figure 4:**
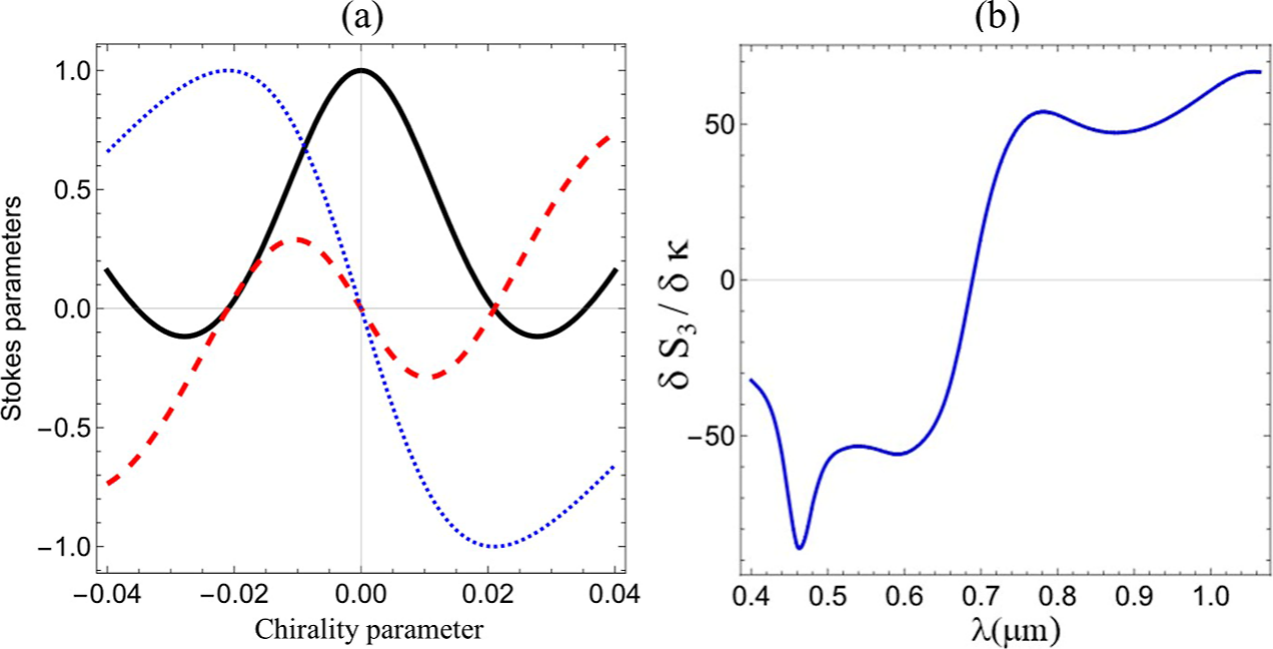
(a) Stokes parameters *S*_1_ (black solid line), *S*_2_ (red dashed line), and *S*_3_ (blue dotted line), normalized by the Stokes parameter *S*_0_, as functions of the microsphere chiral parameter κ for the wavelength λ = 0.464 μm. (b) Slope δ*S*_3_/δκ near κ = 0 as a function of λ. The microsphere radius is 1.5 μm and the NA is NA = 1.3 for both panels.

Besides demonstrating detectable values of the Stokes parameter *S*_3_ from the light scattered by chiral lossless spheres, it is important to compare these values to the Stokes parameter *S*_2_, which gives an optical rotatory power. At first glance, one could argue that *S*_2_ should always dominate over *S*_3_ due to the fact that the particle is lossless, regardless of the detection setup, wavelength λ, and the value of NA. However, Figure 5, where the ratio |*S*_3_/*S*_2_| is calculated as a function of κ and λ (panel (a)) and of κ and NA (panel (b)), demonstrates that this is not true. In fact, |*S*_3_| can be ten times larger than |*S*_2_| for a broad, nonresonant range of wavelengths and values of NA. In contrast, with single chiral plasmonic particles, large values of CD are typically achieved in a narrow frequency window due to a plasmon resonance, which is unavoidably associated with detrimental losses.^39–41^ For lossless spheres *S*_3_ can be even 2 orders of magnitude higher than *S*_2_ at specific Mie resonances with high quality factors, which do not imply losses. Figure 5a,b corroborates the previous results that disclose the conditions for the existence of a sizable CD-like effect for lossless chiral spheres, namely, the Mie regime (*a* ≃ λ) and large NA, respectively. The white regions in Figure 5 correspond to scenarios where *S*_2_ dominates over *S*_3_. In such cases, optical rotatory power is expected to be a more suitable metric for characterizing the chiroptical response of a single chiral particle. Ideally, Figure 5 serves as a theoretical roadmap to facilitate more efficient enantioselection and chiral characterization of lossless, isolated chiral spheres—whether this is achieved through analysis of optical rotatory power or a CD-like signal.

**Figure 5:**
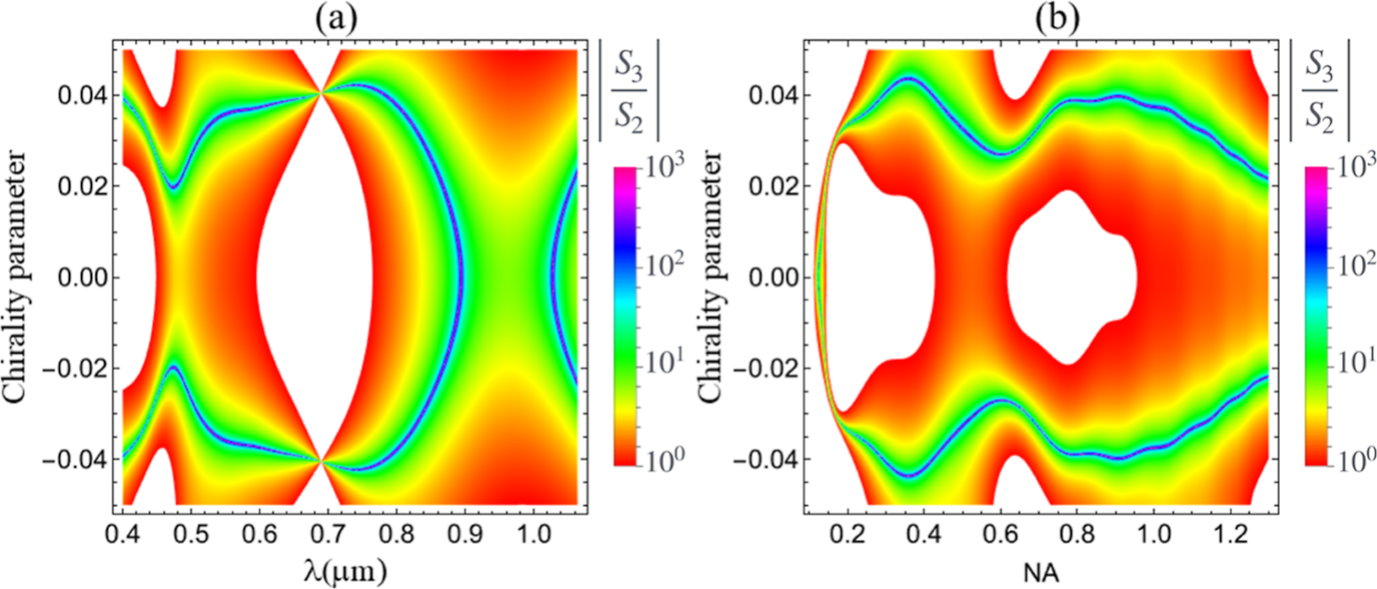
Color maps of |*S*_3_/*S*_2_| (log scale) as a function of the chirality parameter κ and of (a) wavelength λ or (b) NA NA. We take NA = 1.3 for the former and λ = 0.464 μm for the latter. The microsphere radius is 1.5 μm and the regions, where |*S*_3_/*S*_2_| < 1 are white.

Finally, it is important to emphasize that a nonvanishing value of *S*_3_ is not related to any optical anisotropy of the system since the scattering sphere is homogeneous and isotropic. As a result, the Mueller matrix^75^ describing the scattered radiation would capture only genuine CD-like terms.

## Conclusions

In conclusion, we unveil an alternative chiroptical response of all-dielectric Mie chiral particles that is phenomenologically analogous, although neither mathematically nor physically equivalent, to CD, well-known in absorbing media. This phenomenon shows up as large values of the Stokes parameter *S*_3_ for a broad frequency range that we demonstrate to only exist in the Mie scattering regime and for large numerical apertures, an experimentally feasible scenario that nevertheless is not the typical configuration of standard spectrometers, which often use large off-axis detection. By disclosing that chiral Mie particles exhibit an effect that mimics CD, we pave the way for polarimetric applications in Mie resonant metaphotonics (also known as Mie-tronics), where all-dielectric scattering particles substitute traditional plasmonic structures to achieve many practical applications for subwavelength trapping of light.^48^ Our results provide then a link between chiral photonics of single particles and Mie-tronics, enabling potential applications that involve directional scattering with the generation of pure circular polarization states (*S*_3_ = ± 1), corresponding to maximal spin angular momentum transfer, enantioselection, and characterization of the chiroptical response of isolated chiral, lossless particles.

## Supplementary Material



## Notes

Mie coefficients of a chiral microsphere and total detected field; derivation for the optical chirality flux in the Mie scattering scenario (PDF)
