# Supplementary material for: Circular Dichroism without Absorption in Isolated Chiral Dielectric Mie Particles
Source: ACS Photonics. 2026 Feb 9;13(4):949–57. doi: 10.1021/acsphotonics.5c02076 (PMC12922181; doi:10.1021/acsphotonics.5c02076)
Supplement: Supplementary file 1 [file ph5c02076_si_001.pdf]

Supporting Information: Circular Dichroism without absorption in isolated chiral dielectric Mie particles

Rafael S. Dutra,[*,†] Felipe A. Pinheiro,[‡] Diney S. Ether Jr,[‡] Cyriaque Genet,[¶] Nathan B. Viana,[‡] and Paulo A. Maia Neto[*,‡]

†*LISComp-IFRJ , Instituto Federal de Educação, Ciência e Tecnologia, Rua Sebastião de Lacerda, Paracambi, RJ, 26600-000, Brasil*

‡*Instituto de Física, Universidade Federal do Rio de Janeiro, Caixa Postal 68528, Rio de Janeiro, Rio de Janeiro 21941-972, Brazil*

¶ *Université de Strasbourg, CNRS, Institut de Science et d'Ingénierie Supramoléculaires, UMR 7006, F- 67000 Strasbourg, France*

E-mail: rafael.dutra@ifrj.edu.br; pamn@if.ufrj.br

1 Mie Coefficients for a Chiral Sphere

By applying the boundary conditions on the surface of the microsphere of radius a, effective scattering coefficients A_j^σ and B_j^σ associated with the electric and magnetic multipoles are obtained, respectively, and expressed by

$$A_j^\sigma = a_j + i\sigma d_j \quad \textit{and} \quad B_j^\sigma = b_j - i\sigma c_j, \tag{S1}$$

in which the scattering coefficients a_j, b_j, c_j, and d_j are written in terms of the size parameter $x = \omega a/c$ through the following expressions:[1]

$$a_j(x) = \Delta_j^{-1}(x)\left[V_j^{\mathrm{R}}(x)A_j^{\mathrm{L}}(x) + V_j^{\mathrm{L}}(x)A_j^{\mathrm{R}}(x)\right] \tag{S2}$$

$$b_j(x) = \Delta_j^{-1}(x)\left[W_j^{\mathrm{R}}(x)B_j^{\mathrm{L}}(x) + W_j^{\mathrm{L}}(x)B_j^{\mathrm{R}}(x)\right] \tag{S3}$$

$$c_j(x) = i\Delta_j^{-1}(x)\left[W_j^{\mathrm{R}}(x)A_j^{\mathrm{L}}(x) - W_j^{\mathrm{L}}(x)A_j^{\mathrm{R}}(x)\right] \tag{S4}$$

in which we use the following auxiliary functions

$$\Delta_j(x) = W_j^{\mathrm{L}}(x)V_j^{\mathrm{R}}(x) + W_j^{\mathrm{R}}(x)V_j^{\mathrm{L}}(x) \tag{S5}$$

$$W_j^{\mathrm{L,R}}(x) = M\psi_j(N_{\mathrm{L,R}}x)\xi_j'(x) - \xi_j(x)\psi_j'(N_{\mathrm{L,R}}x) \tag{S6}$$

$$V_j^{\mathrm{L,R}}(x) = \psi_j(N_{\mathrm{L,R}}x)\xi_j'(x) - M\xi_j(x)\psi_j'(N_{\mathrm{L,R}}x) \tag{S7}$$

$$A_j^{\mathrm{L,R}}(x) = M\psi_j(N_{\mathrm{L,R}}x)\psi_j'(x) - \psi_j(x)\psi_j'(N_{\mathrm{L,R}}x) \tag{S8}$$

$$B_j^{\mathrm{L,R}}(x) = \psi_j(N_{\mathrm{L,R}}x)\psi_j'(x) - M\psi_j(x)\psi_j'(N_{\mathrm{L,R}}x)\,. \tag{S9}$$

with the relative refractive index $N_{\mathrm{L,R}} = (\sqrt{\epsilon\mu} \pm \kappa)/n_{\mathrm{w}}$ and the relative impedance $M = \sqrt{\mu/\epsilon}\, n_{\mathrm{w}}$. The scattering coefficients are represented using the Riccati-Bessel functions $\psi_j(z) = zj_j(z)$ and $\xi_j(z) = zh_j^{(1)}(z)$. The mixed polarization scattering coefficients satisfy $c_j = -d_j$. In the limit where the chirality parameter approaches zero, the scattering coefficients simplify to the usual Mie coefficients $a_j = A_j/W_j$, $b_j = B_j/V_j$, and $c_j = d_j = 0$.

2 Total Field Detected

Taking Eq. (6) as a starting point and adapting the formalism of Ref.,[2] we find an explicit integral representation for the scattered field helicity components

$$\mathbf{E}_{\mathrm{s,tube}}(\rho_t, \phi_t, z_t) = \sum_{\sigma=-1,+1} E^{\sigma}_{s,\mathrm{tube}}(\rho_t, \phi_t, z_t)\,\hat{\boldsymbol{\varepsilon}}^{\sigma}$$

after propagation through the objective (focal length f) and tube (focal length f') lenses in terms of the cylindrical coordinates (ρ_t, ϕ_t, z_t) of a position at the CCD camera:

$$E^{\sigma}_{s,\mathrm{tube}}(\rho_t, \phi_t, z_t) = \frac{\sqrt{2}E_0}{4}\frac{f}{nf'}e^{ikf}e^{ik_0(D+f'+z_t)}\sum_{j=1}^{\infty}(2j+1)\int_0^{\theta_0} d\theta\, g(\theta)$$

$$\times\Bigg[(A^{\sigma}_j + B^{\sigma}_j)d^j_{\sigma,\sigma}(\alpha)J_0\left(k_0\frac{f}{f'}\rho_t\sin\theta\right)$$

$$+(A^{\sigma}_j - B^{\sigma}_j)e^{-2i\sigma\phi_t}d^j_{-\sigma,\sigma}(\alpha)J_{2\sigma}\left(k_0\frac{f}{f'}\rho_t\sin\theta\right)\Bigg], \tag{S10}$$

where k_0 is the wave number in the tube lens region, J_n denote the cylindrical Bessel functions and $g(\theta) = (\sin\theta/\cos\alpha)(\cos\theta)^{3/2}\, T_{\perp}(\theta)e^{i\Phi_{\mathrm{g-w}}(\theta)}e^{-ik_0z_t\frac{f^2}{2f'^2}\sin^2\theta}$. In the situation where detection is carried out at the focus of the tube lens, with $\rho_t = 0$ and $z_t = 0$, we derive Eq. (8) from (S10) by using that $J_n(0) = \delta_{n0}$.

Note that for an achiral sphere ($\kappa = 0$), the Mie coefficients become $A^{\sigma}_j \to a_j$ and $B^{\sigma}_j \to b_j$, with a_j and b_j denoting the standard electric and magnetic Mie coefficients,[3] respectively. They no longer depend on σ, and both helicities are scattered with the same amplitude. In this case, the summation over σ in Eq. (8) is simplified as the only term depending on helicity is the polarization unit vector itself. We then sum over σ and find that the linear polarization of the incident field is conserved:

$$\mathbf{E}^{\mathrm{achiral}}_{s,\mathrm{tube}} = \frac{E_0}{2}\frac{f}{n_g f'}e^{ik_gf}e^{ik_0(D+f')}\sum_{j=1}^{\infty}(2j+1)(a_j+b_j)\int_0^{\theta_0} d^j_{1,1}(\alpha)f(\theta)d\theta\,\hat{\mathbf{x}}. \tag{S11}$$

In turn, the illumination field propagated through the objective and tube lenses is expressed as

$$\mathbf{E}_{\mathrm{in,tube}}(\mathbf{r}_t) = -\frac{E_0 f}{f'}\frac{2n_{\mathrm{w}}}{n_{\mathrm{w}}+n_g}e^{i(k_{\mathrm{w}}L_c - k_gL_g)}e^{ik_0(f'+D)}e^{ik_gf}\,\hat{\mathbf{x}}. \tag{S12}$$

3 Optical chirality Flux in the Mie Scattering Scenario

In this section, we verify the conservation law for the optical chirality flux[4] in the scenario of scattering by a Mie sphere immersed in a homogeneous medium.

3.1 Chirality Flux

Using Maxwell's equations in the absence of sources, the optical chirality flux density can be expressed as

$$\mathbf{F} = \frac{i\omega}{4}(\varepsilon^{*}\mathbf{E}\times\mathbf{E}^{*} - \mu\,\mathbf{H}^{*}\times\mathbf{H}). \tag{S13}$$

We evaluate the time-averaged chirality flux Φ over an imaginary sphere of infinite radius, enclosing the Mie sphere, in terms of the total fields given by the superposition of incident and scattered fields $\mathbf{E}_{tot} = \mathbf{E}_{in} + \mathbf{E}_{s}$ and $\mathbf{H}_{tot} = \mathbf{H}_{in} + \mathbf{H}_{s}$:

$$\Phi = \Re\left(\oint_{s} \mathbf{F}\cdot\hat{r}\,dS\right). \tag{S14}$$

It is convenient to expand the total electromagnetic field $(\mathbf{E}_{tot}, \mathbf{H}_{tot})$ in spherical waves using the electric (E) and magnetic (M) multipoles, $\mathbf{E}_{tot} = ik(\,\mathbf{I}_{z}^{E} - (\mu\,c/n_1)\mathbf{I}_{x}^{M}\,)$ and $\mathbf{H}_{tot} = ik(\,\mathbf{I}_{z}^{M} + (\varepsilon\,c/n_1)\mathbf{I}_{x}^{E}\,)$:

$$\mathbf{I}_{z}^{E,M} = i\frac{C^{E,M}}{kr}\sum_{JM}\Gamma_{J,M}^{E,M}(kr)(i)\left(\,\hat{r}\times\mathbf{L}(\,Y_{J,M}(\theta,\phi)\,)\,\right) \tag{S15}$$

$$\mathbf{I}_{x}^{E,M} = C^{E,M}\sum_{JM}\Omega_{J,M}^{E,M}(kr)(i)\mathbf{L}(\,Y_{J,M}(\theta,\phi)\,), \tag{S16}$$

where $\mathbf{L} = -i\mathbf{r}\times\boldsymbol{\nabla}$. We keep only the tangential components of the fields, since the radial components do not contribute to the flux in the radiation zone. The scattered amplitudes and the radial dependence are taken into account implicitly in functions $\Gamma_{J,M}^{E,M}(kr)$ and $\Omega_{J,M}^{E,M}(kr)$. Substituting eq. S13 into S14, together with the fields, we obtain:

$$\Phi = -\frac{\omega\, k^2}{4}\Im\Bigg[\oint_s \Bigg(\varepsilon^*(\mathbf{I}_z^E \times \mathbf{I}_z^{E*})\cdot\hat{r} - \mu\,(\mathbf{I}_z^{M*}\times\mathbf{I}_z^M)\cdot\hat{r}\Bigg)\,dS\Bigg] +$$

$$-\frac{\omega\, k^2}{4}\Im\Bigg[\oint_s \Bigg(\frac{\varepsilon^*\mu^2 c^2}{n_1^2}(\mathbf{I}_x^M \times \mathbf{I}_x^{M*})\cdot\hat{r} - \frac{\mu\,\varepsilon^2 c^2}{n_1^2}(\mathbf{I}_x^{E*}\times\mathbf{I}_x^E)\cdot\hat{r}\Bigg)\,dS\Bigg]$$

$$+\frac{\omega\, k^2}{2}\Im\Bigg[\oint_s \Bigg(\varepsilon^*\Re\Bigg(\frac{\mu^* c}{n_1^*}(\mathbf{I}_z^E \times \mathbf{I}_x^{M*})\cdot\hat{r}\Bigg) - \mu\,\Re\Bigg(\frac{\varepsilon c}{n_1}(\mathbf{I}_z^{M*}\times\mathbf{I}_x^E)\cdot\hat{r}\Bigg)\Bigg)\,dS\Bigg] \qquad \text{(S17)}$$

According to equation S17, two types of terms, quadratic and cross terms in the multipoles, contribute to the chirality flux.

3.2 Quadratic Terms

The first two terms in equation S17 involve surface integrals of the type $I = \oint_s (\mathbf{I}_z^{E,M} \times \mathbf{I}_z^{E,M*})\cdot \hat{r}\, dS$. Using equation S15, we obtain

$$I = -\frac{C^{E,M^2}}{k^2 r^2}\sum_{JM}\sum_{J'M'}\Gamma_{JM}^{E,M}\Gamma_{J'M'}^{E,M*}\oint_s \Bigg(\hat{r}\times\mathbf{L}(\,Y_{J,M}(\theta,\phi)\,)\Bigg)\times\Bigg(\hat{r}\times\mathbf{L}(\,Y_{J',M'}^*(\theta,\phi)\,)\Bigg)\cdot\hat{r}dS. \qquad \text{(S18)}$$

Using $dS = r^2 sin\theta d\theta d\phi$, $\mathbf{L} = -(i)\Bigg(\hat{\phi}\,\partial_\theta Y(\theta,\phi) - \hat{\theta}\,\partial_\phi Y(\theta,\phi)/\sin\theta\Bigg)$, $\partial_\phi Y_{J,M}(\theta,\phi) = iMY_{J,M}(\theta,\phi)$ and $\int_0^{2\pi} e^{i(M'-M)\phi}\, d\phi = 2\pi\delta_{M,M'}$, we find that

$$I = 2\pi\, ir^2\frac{C^{E,M^2}}{k^2 r^2}\sum_{JM}\sum_{J'}\Gamma_{JM}^{E,M}\Gamma_{J'M}^{E,M*}M\int_0^\pi \frac{\partial}{\partial\theta}\Bigg(Y_{J,M}(\theta,0)Y_{J',M}^*(\theta,0)\Bigg)\,d\theta = 0 \qquad \text{(S19)}$$

Similarly, it can be shown that the quadratic terms $\oint_s(\mathbf{I}_x^{E,M}\times\mathbf{I}_x^{E,M*})\cdot\hat{r}\,dS$ also do not contribute to the flux. For a homogeneous medium, the quadratic terms in the electric and magnetic multipoles do not contribute to the chirality flux.

3.3 Cross Terms

The contribution to the chirality flux comes only from the cross terms in the multipoles (E) and (M) in the situation of a homogeneous host medium with absorption:

$$\Phi = \frac{\omega\, k^2}{2}\left[\oint_s \left(\Im(\varepsilon^*)\Re\left(\frac{\mu^* c}{n_1^*}(\mathbf{I}_z^E \times \mathbf{I}_x^{M*})\cdot \hat{r}\right) - \Im(\mu)\,\Re\left(\frac{\varepsilon c}{n_1}(\mathbf{I}_z^{M*} \times \mathbf{I}_x^{E})\cdot \hat{r}\right)\right) dS\right]. \quad \text{(S20)}$$

For a host medium without absorption, $\Im(\varepsilon) = \Im(\mu) = 0$, and the chirality flux through the sphere vanishes in agreement with the general result of Ref.[4]

References

(1) Bohren, C. F. Light scattering by an optically active sphere. *Chemical Physics Letters* **1974**, *29*, 458–462.

(2) Gómez, F.; Dutra, R.; Pires, L.; Araújo, G. R. d. S.; Pontes, B.; Neto, P. M.; Nussenzveig, H.; Viana, N. Nonparaxial Mie theory of image formation in optical microscopes and characterization of colloidal particles. *Physical Review Applied* **2021**, *15*, 064012.

(3) Bohren, C. F.; Huffmann, D. R. *Absorption and Scattering of Light by Small Paricles*; Wiley-VCH, 1983.

(4) Poulikakos, L. V.; Gutsche, P.; McPeak, K. M.; Burger, S.; Niegemann, J.; Hafner, C.; Norris, D. J. Optical chirality flux as a useful far-field probe of chiral near fields. *ACS photonics* **2016**, *3*, 1619–1625.
